# Novel *GPR143* mutations and clinical characteristics in six Chinese families with X-linked ocular albinism

**Published:** 2008-10-30

**Authors:** Shaohua Fang, Xiangming Guo, Xiaoyun Jia, Xueshan Xiao, Shiqiang Li, Qingjiong Zhang

**Affiliations:** State Key Laboratory of Ophthalmology, Zhongshan Ophthalmic Center, Sun Yat-sen University, Guangzhou, People’s Republic of China

## Abstract

**Purpose:**

There are few genetic studies and clinical descriptions of Asian patients with X-linked ocular albinism (OA1). In the present study, the mutation analysis of *G protein-coupled receptor 143 gene (GPR143)* and clinical characteristics were assessed in Chinese patients with OA1.

**Methods:**

Six families with OA1 were recruited from our pediatric and genetic eye clinic. Genomic DNA was prepared from venous leukocytes. The coding regions of *GPR143* were amplified by polymerase chain reaction, and subsequently analyzed by direct sequencing. The variations detected were further evaluated in available family members as well as controls.

**Results:**

Mutations in *GPR143* were identified in each of the six families: c.849delT (p.Val284SerfsX15); c.238_240delCTC (p.Leu80del); c.658+1G>A, c.353G>A (p.Gly118Glu); g.1103_7266del6164 (p.Gly84AlafsX65), which resulted in a deletion of exons 2 and 3; and g.25985_26546del562 (p.Gly296ValfsX26), which resulted in a deletion of exon 8. Of these six, c.353G>A is a known mutation, while the other five are novel. All affected patients had nystagmus, poor visual acuity, and foveal hypoplasia. However, hypopigmentation of the iris and fundus was very mild in these patients.

**Conclusions:**

Five novel mutations and one known mutation were identified in six Chinese families with OA1. These results expand the mutation spectrum of *GPR143*, and demonstrate the clinical characteristics of OA1 among the Chinese.

## Introduction

X-linked ocular albinism (OA1; OMIM 300500), also called ocular albinism type 1, is the most common form of ocular albinism. Most affected white males exhibit nystagmus, poor visual acuity, iris translucency, foveal hypoplasia, an albinotic fundus, and normally pigmented skin and hair [1-4]. Other findings may include photophobia, strabismus, and misrouting of the optic tracts, resulting in loss of stereoscopic vision [5-7]. In some OA1 patients of black people, O'Donnell found hypopigmentation of the iris and retina may be subtle, such that these patients present a nonalbinotic fundus and little iris translucency [8]. However, the characteristics of OA1 have not been well defined in Asians. The misdiagnosis of OA1 as congenital nystagmus is not uncommon, and is usually due to lack of clinical experience or lack of phenotypic characterization for certain ethnic groups [9-14]. Such misdiagnoses might have gone undetected before the OA1 gene was identified.

OA1 is caused by mutations in the *G protein-coupled receptor 143 (GPR143)* gene (OMIM 300500), originally also known as the OA1 gene, located at Xp22.32 [15-17]. Various types of mutations in *GPR143* have been identified in Caucasians living in the Netherlands, the United Kingdom, the United States, Germany, Canada, Belgium, France, Italy, and South Africa (albinism). Interestingly, genetic studies of Chinese patients with OA1 are rare.

In this study, sequence analysis of *GPR143* resulted in the identification of mutations in each family, including five novel and one known mutations. Furthermore, we describe the clinical characteristics of OA1 patients and female carriers from six Chinese families.

## Methods

### Patients

**Figure 1:**
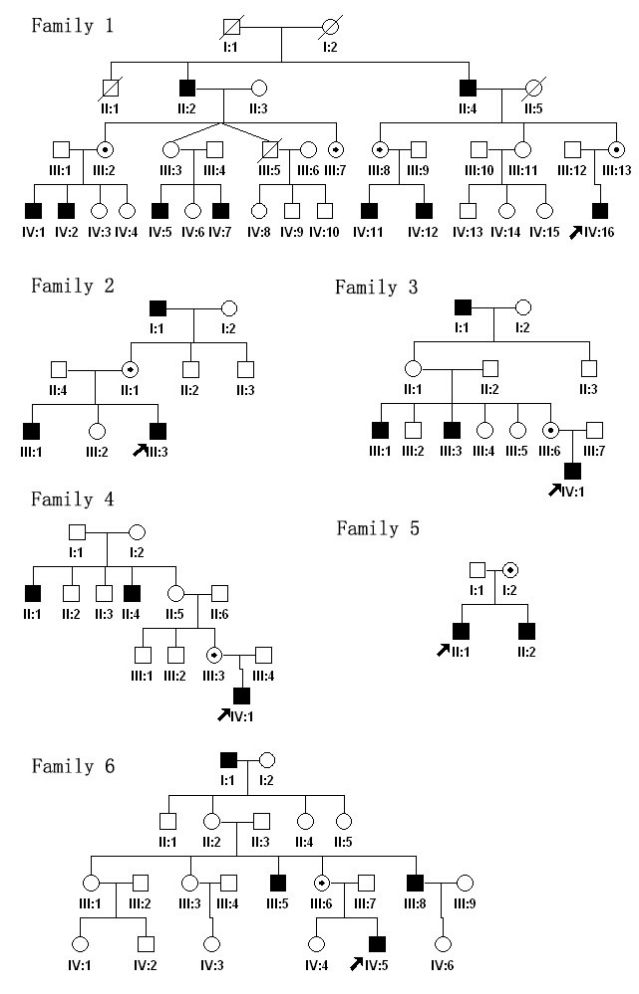
Pedigrees of the six families are shown. Black filled symbols indicate patients affected with OA1 in each family. Dot-marked symbols represent carriers. The proband is marked by arrow in each family.

There are 15 patients and 7 carriers participated in this study (Figure 1, Table 1), further more, 100 normal male controls mainly from the South of China also take part in, all these people are collected by our Pediatric and Genetic Clinic of Zhongshan Ophthalmic Center. Informed consent conforming to the tenets of the Declaration of Helsinki was obtained from each participant before the study. Medical and ophthalmic histories were obtained. Ophthalmological examination including visual acuity assessment , slit lamp biomicroscopic and ophthalmoscopic observation was performed by Drs. Guo and Zhang. Anterior and posterior segments of the eyes were documented by slit lamp and fundus photography. Flash visual evoked potential (VEP) was performed in one 4-year-old patient (proband in family 3). Genomic DNA was prepared from leukocytes of peripheral venous blood using the phenol-chloroform extracted method.

**Table 1:** Summary of clinical findings in affected males and carriers

**Family**	**ID#patients**	**Gender**	**Age (yrs)**	**First symptom**	**VA (corrected)**	**Iris hypopigmentation**	**Fundus a,b,c**	**Nys**
1	IV16	M	8	nystagmus	0.2/0.2	normal	no,no, yes	yes
	IV5	M	27	nystagmus	0.25/0.3	mild	no,no,yes	yes
	IV7	M	23	nystagmus	0.3/0.2	mild	no,no,yes	yes
	IV2	M	24	nystagmus	0.2/0.25	normal	no,no,yes	yes
	II4	M	64	nystagmus	0.4/0.2	normal	no,yes,yes	yes
	II2	M	77	nystagmus	0.12/0.06	normal	no,yes,yes	yes
	IV11	M	11	nystagmus	0.3/0.3	normal	no,no,yes	yes
	IV12	M	9	nystagmus	0.2/0.12	normal	no,no,yes	yes
2	III3	M	4	poor VA	0.1/0.1	mild	ND	yes
	III1	M	6	poor VA	0.2/0.2	obvious	no,no,yes	yes
3	IV1	M	4	poor VA	ND	mild	no,no,yes	yes
4	IV1	M	7	nystagmus	0.2/0.2	mild	no,yes,yes	yes
5	II1	M	12	nystagmus	0.2/0.2	normal	no,no,yes	yes
	II2	M	7	nystagmus	0.2/0.3	normal	no,no,yes	yes
6	IV5	M	4	nystagmus	ND	mild	no,yes,yes	yes
Carriers								
1	III13	F	38	NA	0.8/1.0	normal	no,no,no	NA
	III7	F	41	NA	0.8/1.0	normal	no,no,no	NA
	III8	F	36	NA	1.0/1.0	normal	no,no,no	NA
	III2	F	50	NA	1.0/1.0	normal	no,no,no	NA
3	III6	F	31	NA	1.2/1.2	mild	no,yes,no	NA
4	III3	F	33	NA	1.0/1.0	mild	no,yes,no	NA
6	III6	F	31	NA	1.0/1.0	normal	no,yes,no	NA

Clinical characteristics including gender, age, first symptom, visual acuity, iris, and fundus hypopigmentation form 15 OA1 patients and 7 carriers were conclude in this table. Abbreviations: albinotic fundus (a); hypopigmentation (b); foveal hypoplasia (c); not determined (ND); not applicable (NA), visual acuity(VA); nystagmus (Nys).

### Mutation analysis

Eleven pairs of primers (Table 2) were used to amplify the nine coding exons and the adjacent intronic sequences of *GPR143* (NCBI human genome build 36.2, GeneID 4935, NC_000023.9 for gDNA, NM_000273 for mRNA, NP_000264 for protein). Sequence analysis was performed as previously described [18]. The numbering system of the nucleotides for 404 amino acids, but not 424, was used for mutation description as previously described [19] (see in “In silico analysis” part).

**Table 2:** Primers for amplifying and sequencing *GPR143* genomic segments.

**Exon**	**Forward primer (5′- 3′)**	**Reverse primer (5′- 3′)**	**Product size (bp)**	**Annealing temperature(°C)**
1A	CTCCTCCGCCCGCCCAAGCATCAC	CCCAGGCAGCCGAGAAGGTC	464	68
1B	CCGCGCCTAGGGACCTTCTGCT	AACCCGCGGGCCTCTCGTCCTCAC	399	70
2	CTTTCTTCCTTTTCCCTCCTTGTC	GTTTGCTGCTGCTGCGATTTG	360	58
3	CACGTGCGGCTTCCTGAC	TTGGCCTCTTATAAAAATGA	385	56
4	GGGCTTTCCTCTGTGTACATTTTC	CCCTGAGACAACGGCCTAACC	334	60
5	GCATTTCCCTTTTTGTTCTCATCC	AGGCCTGCACATTTTCATTTATTG	406	58
6	TTGCTTCCTGCCCCTCTGG	ACTTGCTCCCCTGTCCTCTGT	400	60
7	TGCACCTGGCCCTCTTAGTTTC	TCAGGAGGCCAAGACAGAGGAT	441	60
8A	AAACCAACCCACCAACCAGTCAAC	GCATGCTCAGGGCTTCGTCA	395	60
8B	CCAGCCCAGGGATTTCTCTT	ACCCCGCCATGCACAGGAC	329	60
9	AGCTGATGACAAACCTGCTAG	CCCTTTCTCCTATCCTAAAG	330	58
FAM5	CCTAGGGACCGGATGGGACAAC	CTCGCCAACAGTTACACAGCTC	487	60
FAM6	GTCCCCTTTCTTCCCTTCTCTACT	GTGGTGTTTGTCTTTGGTTTTGTG	297	60
SSCP-1	AGCCACGCAGCTCGTGCTGAGCTTCCAGCC	CCCAGGCGCTGATCAGATTCCAACCCGCGG	250	70
SSCP-2	CATCCTCTTATCTTGACTTCC	CCCAGAGAGCTTCCCTAGT	224	58
SSCP-3	CATGTTCTCTTTACCTGCTGC	AGTGAGACCTTGTCTCTGAAG	227	56

Listed are primer sequences, sizes of PCR products, and annealing temperature used for the amplification. Primers 1-9 were used to amplify and sequence the *GPR143* genimic segments. Primers FAM5 and FAM6 were used to detect the deletion boundaries in families 5 and 6, respectively. Primers *SSCP-1* to 3 were used to amplify exons 1, 5, and 7 for heterduplex-SSCP analysis.

Any variations detected, except for two large intragenic deletions, were further evaluated in available family members as well as in 100 Chinese normal male controls using heteroduplex-single strand conformation polymorphism (SSCP) analysis, as previously described [20]. Three additional pairs of primers (Table 2, available on request) [21] were synthesized for heteroduplex-SSCP analysis of exons 1, 5, and 7.

In family 5, no PCR products were produced for the DNA fragments encompassing exons 2 and 3 of *GPR143*, suggesting a deletion in this region. To define the boundaries of the deletion, we designed a series of primer pairs based on sequences between exons 1 and 4 to amplify junctional fragments harboring the deletion. The deletion boundary was finally identified using the primer pairs shown in Table 2 (FAM5). In family 6, no PCR product was detected for exon 8. We used the same technique to find the deletion boundary with the primer pairs FAM6 shown in Table 2.

### In silico analysis

Direct sequencing of the PCR products was performed with an ABI BigDye Terminator Cycle Sequencing Kit v3.1 (ABI Applied Biosystems, Foster City, CA) using an ABI3100 Genetic Analyzer. Sequencing results from patients as well as *GPR143* consensus sequences from the NCBI human genome database (NM_000273) were imported into the SeqManII program of the Lasergene package (DNAStar Inc., Madison, WI) to identify variations. Each mutation was confirmed by bidirectional sequencing. Mutation descriptions followed the nomenclature recommended by the Human Genomic Variation Society (HGVS). To evaluate the splice site mutation, we used the Automated Splice Site Analysis (ASSA) server [22] to analyze the possible effects of this kind of mutation.

## Results

### Clinical phenotype

**Figure 2:**
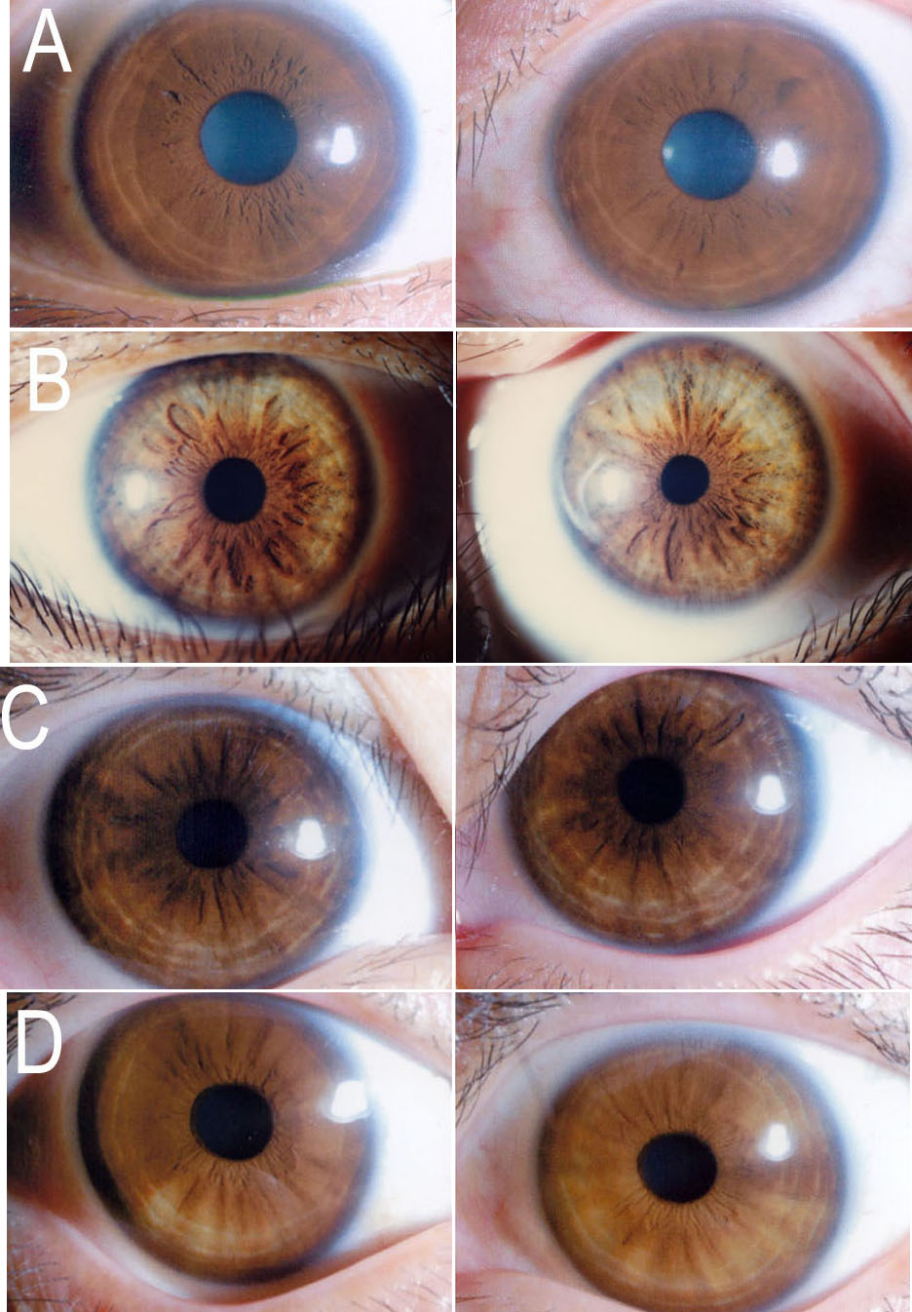
Photographs of irises from normal control, patients, and carriers. Both of eyes′ irises are presented. **A** is a normal person whose irises exhibited normal pigmentation. **B** shows the irises of proband (III:3) in family 2. He is the only one patient who had obvious iris hypopigmentation. Obvious hypopigmentation is observed in both of eyes in picture **B**. **C** is the proband (IV:1) of family 4, showing slightly hypopigmentation in peripheral of the both eyes. **D** is carrier (III:3) in family 4. Her iris hypopigmentation is also very mild in both of eyes.

**Figure 3:**
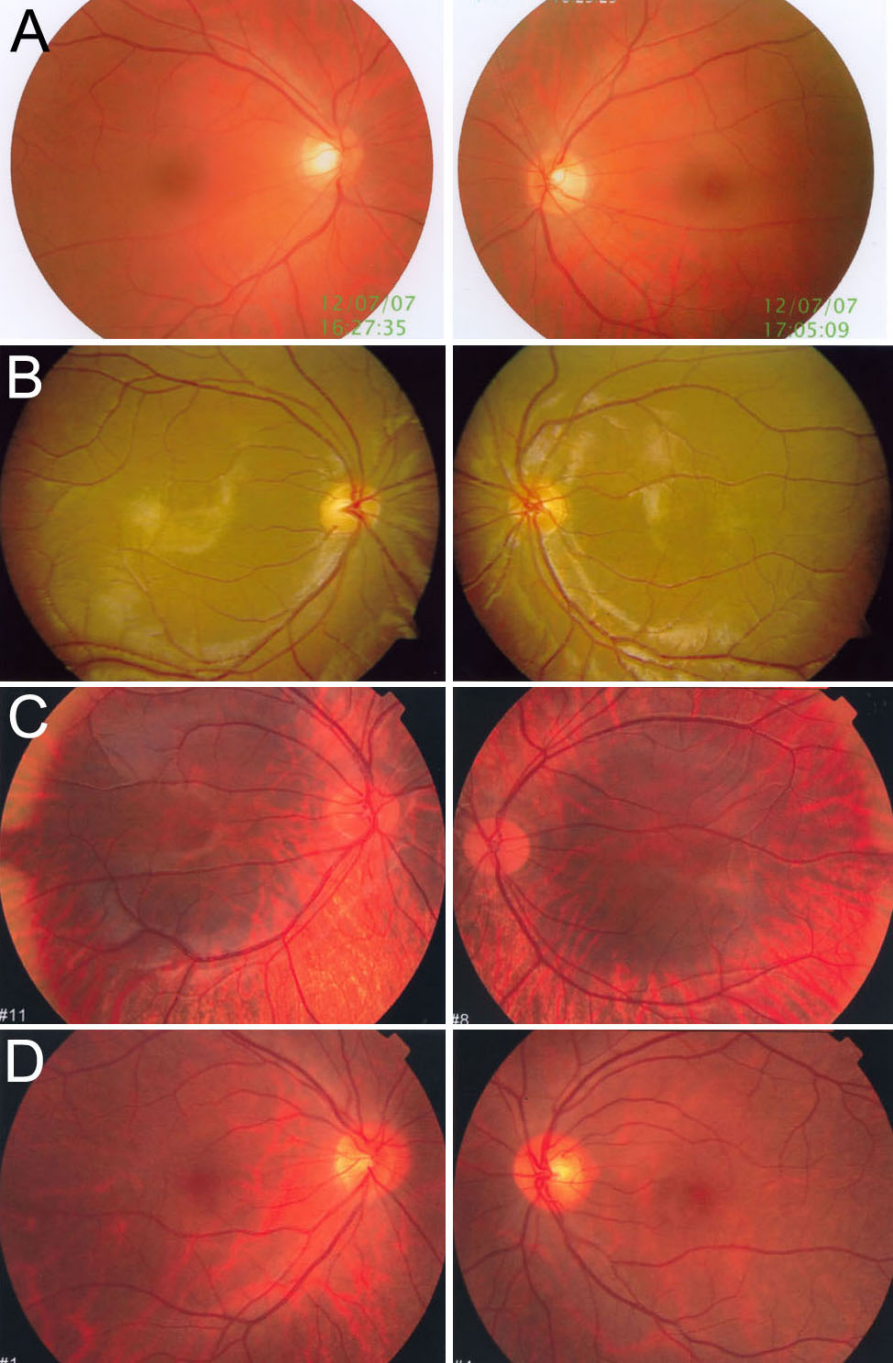
Photographs of fundi from normal control, patients, and carriers. Both of eyes′ fundi are presented. **A** is the fundi from a normal control. **B** shows the fundi of proband (IV:16) in family 1. There is normal pigmentation and severe foveal hypoplasia in both of eyes in picture **B**. **C** presents the fundi of proband IV:1 from family 4. There was hypopigmentation in the posterior of the fundus and severe foveal hypoplasia in both of eyes in picture **C**. **D** shows the fundi from carrier III:3 in family 4. There was pigmentary mosaicism in the retinal pigment epithelium, and the fovea was normal in both of eyes.

The clinical characteristics of OA1 in Chinese individuals are described as in Table 1 and Figure 2 and Figure 3. Table 1 shows the gender, age and clinical characteristics of the 15 patients and 7 carriers from the six families. Figure 2 and Figure 3 show some of the patients and carriers′ iris and fundus image respectively.

#### Reduced visual acuity and nystagmus

All OA1 patients who came to our ophthalmic center had nystagmus and poor visual acuity as a first symptom. The nystagmus was present since birth, and corrected visual acuity (VA) was between 0.1–0.3. No VA data were obtained for families 3 and 6 because the patients were not old enough (Table 1) to cooperate with the examination. Flash VEP was performed for the family 3 proband, and no abnormalities were observed. The carriers did not show any symptoms, and their VA was normal (Table 1). All participants studied had normal skin and hair.

#### Iris hypopigmentation

This study examined 15 Chinese OA1 patients. Compared with normal individuals (Figure 2A), only one patient had obvious iris hypopigmentation (Figure 2B). Six patients exhibited mild peripheral iris hypopigmentation, as shown in Figure 2C, and eight patients had no abnormalities. Of the OA1 carriers, two exhibited mild peripheral iris hypopigmentation that could easily have been overlooked (Figure 2D), while the other carriers exhibited iris pigmentation that was similar to that in normal individuals.

#### Hypopigmentation in the fundus and foveal hypoplasia

This study examined the fundi of 13 patients'. Compared with normal individuals (Figure 3A), eight patients had normal pigmentation Figure 3B, three had mild hypopigmentation, and only two exhibited an albinotic fundus (Figure 3C). However, all of the patients had severe foveal hypoplasia (Table 1). In the proband of family 1 (Figure 3B), there was no typical albinotic fundus, and the fundus pigmentation had no abnormalities compared with normal individuals (Figure 3A). The only sign of the disease we observed was foveal hypoplasia. In the proband of family 4 (Figure 3C), hypopigmentation was apparent in the posterior of the fundus, and foveal hypoplasia was evident. Of the OA1 carriers, some had pigmentary mosaicism in the retinal pigment epithelium—for example, carrier III:3 in family 4 (Figure 3D)—while others had no hypopigmentation (Table 1).

### Mutation analysis

**Figure 4:**
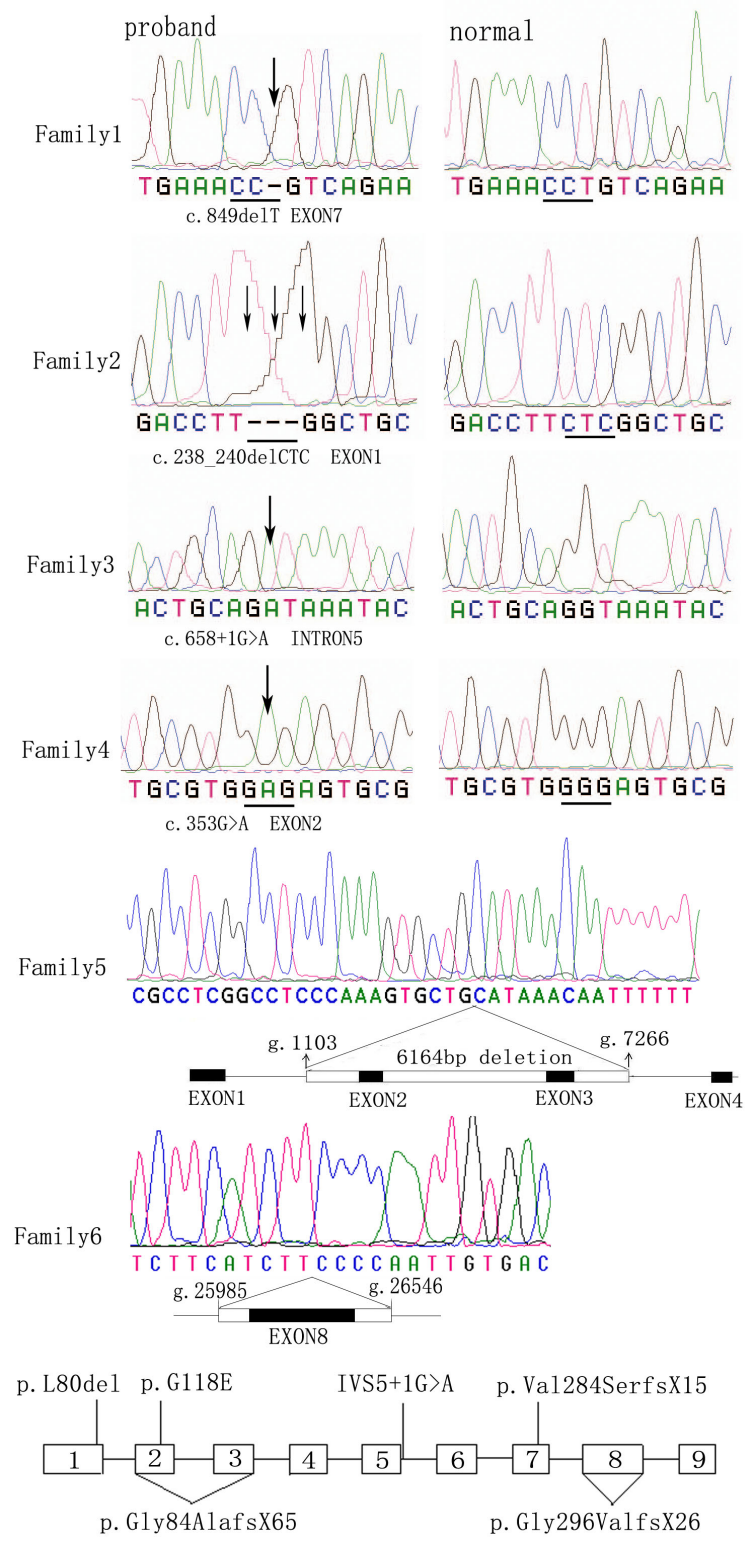
Sequencing analysis of six families with OA1. The mutant *GPR143* sequence (left) and corresponding normal sequence (right) are shown for families 1–4. The exact mutation is labeled under each sequence according to the nomenclature recommended by Human Genomic Variation Society (HGVS). Each mutation is marked with an arrow. In family 5, direct sequencing image was obtained by direct sequencing. The long band in Family 5 including exons 2 and 3, represents the large intragenic deletion region, which encompasses about 6,164 bp from g.1103 to g.7266 of *GPR143*. In family 6, direct sequencing image was obtained by direct sequencing, and the lower long band represents the deletion from g.25985 to g.26546 encompassing exon 8. The location of the six mutations within *GPR143* is exhibited at the bottom of the Figure 4. Exons are shown as open boxes numbered 1–9, and thin lines represent introns. The locations of the six mutations found in this study are indicated beside the exons.

Sequence analysis of *GPR143* detected six mutations among the six families (Table 3, Figure 1, and Figure, 4). Each of the six families studied had one of the following mutations: c.849delT (p.Val284SerfsX15); c.238_240delCTC (p.Leu80del); c.658+1G>A; c.353G>A (p.Gly118Glu); g.1103_7266del6164 (p.Gly84AlafsX65), which resulted in a deletion of exons 2 and 3; and g.25985_26546del562 (p.Gly296ValfsX26), which resulted in a deletion of exon 8. Of these six, c.353G>A is a known mutation, and the other five are novel. These mutations were present in affected hemizygous males and obligate heterozygous female carriers in the families, but were not detected in unaffected males and 100 controls by heteroduplex-SSCP analysis. The locations of the six mutations in *GPR143* are shown in Figure 4.

**Table 3:** *GPR143* mutations identified in six Chinese families with OA1

**Patients**	**Nucleotide change**	**Exon/intron**	**Effect**
Family 1	c.849delT	e7	p.Val284SerfsX15
Family 2	c.238_240delCTC	e1	p.Leu80del
Family 3	c.658+1G>A	i5	Loss of splicing acceptor
Family 4*	c.353G>A	e2	p.Gly118Glu
Family 5	g.1103_7266del6164	Loss of exons 2 and 3	p.Gly84AlafsX65
Family 6	g.25985_26546del562	Loss of exons 8	p.Gly296ValfsX26

Five novel and one known mutations were identified in six Chinese families. The mutation was named according to the nomenclature recommended by Human Genomic Variation Society (HGVS). In the third column, i5 stands for intron 5. The asterisk indicates that the mutation has been previously reported.

The c.849delT mutation (Figure 4, Table 3) is thought to induce a frameshift, creating a termination at codon 892. The resulting gene product lacks about a fourth of its amino acids, including the last transmembrane domain of the C-terminus. The c.238_240delCTC mutation resulted in a leucine deletion in the 80th amino acid position, and the c.353G>A mutation resulted in a replacement of glutamic acid with glycine in the 118th amino acid positions. The splice site mutation, c.658+1G>A, was expected to result in a loss of the corresponding splice sites (Figure 4, Table 3). The ASSA server [22] result predicted that the c.658+1G>A mutation would significantly change the strength of the splicing acceptor (from 9.2 bits to –3.6 bits) and would therefore abolish the natural splice site, corresponding to an approximately 594.6 fold decrease in the predicted affinity for this site. Two large intragenic deletions, g.1103_7266del6164 and g.25985_26546del562, resulted in the loss of an entire exon and produced what we believe to be a nonfunctional protein.

## Discussion

In our study, we found five novel mutations and one known mutation in *GPR143* among six unrelated Chinese families with OA1. All of the patients exhibited congenital nystagmus, poor visual acuity, mild hypopigmentation of the iris and fundus, and severe foveal hypoplasia.

Previous studies indicate that there is a higher detection of mutations in *GPR143* in OA1 patients. Schiaffino et al. [2] screened the entire *GPR143* coding sequence and detected mutations in one-third of patients. Schnur et al. [3] reported a higher frequency (90%) of mutations in their OA1 patient group. Here, our result indicate that Chinese OA1 patients also have a high frequency of mutations in *GPR143* and further confirm that *GPR143* is the major locus for OA1. Based on the high detection of mutations in *GPR143*, molecular genetic testing and prenatal diagnosis should be made available to Chinese OA1 patients.

Of the six mutations in our OA1 patients, there were four deletion mutations, one missense mutation, and one splice site mutation. These mutations mainly cluster within exons 1, 2, 3, and 7 of *GPR143* (Figure 4), and the locations of the mutations are similar to previous studies. Large intragenic deletion is a common mutation in *GPR143*. Bassi et al. [23] found a diverse prevalence of large deletions between European (<10%) and North American (>50%) patients with OA1. Interestingly we also found two large intragenic deletions in our OA1 patients. These results may indicate that large intragenic deletions are also common in Chinese OA1 patients. The c.353G>A (p.Gly118Glu) mutation, resulting in the replacement of glutamic acid with glycine at the 118th amino acid position, has been identified in Caucasian populations [3] and patients from the Netherlands [23]. And this is the third time to report it, the mutation appeared in different ethnic groups indicate that c.353G>A (p.Gly118Glu) may be a hot spot mutation. Our result also shows that Chinese OA1 patients may have a similar spectrum of mutation in *GPR143* compared with the White group. This mutation may therefore be a hot spot mutation in different ethnic groups. The findings from our study show that Chinese OA1 patients may have a similar spectrum of mutation in *GPR143* compared with the Caucasian group.

The OA1 patients in our study exhibited congenital nystagmus and poor visual acuity with mild hypopigmentation of the iris and fundus. Liu et al. [12] reported a large X-linked recessive inheritance in a Chinese family with nystagmus as a prominent and consistent manifestation of the phenotype. None of the patients in the family had the complete classical phenotype of ocular albinism, and patients were initially misdiagnosed with congenital nystagmus. The disease gene was mapped to a region approximately 10.6 Mb in size, flanked by DXS996 and DXS7593 on Xp22 with a significant peak multipoint LOD score. Analysis of 21 candidate genes in the region revealed a novel p.S89F mutation in the second transmembrane domain of *GPR143*.

In our OA1 patients, iris hypopigmentation ranged from mild to normal, with the exception of one patient. It was difficult to distinguish any differences compared to normal individuals. Our OA1 patients showed little change in clinical characteristics of the fundus, exhibiting only mild hypopigmentation and sometimes even normal pigmentation. Severe foveal hypoplasia was the prominent clinical feature observed in Chinese OA1 patients, which may be a useful criterion for distinguishing OA1 from nystagmus of unknown cause. As hypopigmentation of the iris and fundus is not typical among the Chinese, OA1 could easily be misdiagnosed as another disease due to the presence of nystagmus and severely reduced visual acuity. Although foveal hypoplasia may be a key indicator of OA1, unawareness of the clinical characteristics of OA1 and lack of experience might explain why OA1 is rarely reported in the Chinese population. Here, we describe the characteristic signs of OA1 in Chinese patients, potentially facilitating the diagnosis of OA1 in this population. For patients presenting with nystagmus and reduced visual acuity, careful evaluation of iris color, fundus color, and foveal development is critical. Furthermore, molecular genetic testing of *GPR143* might be useful for establishing a diagnosis of OA1.

In summary, this report identified five novel mutations and one known mutation in *GPR143* of Chinese OA1 patients. Moreover, we examined the clinical features of Chinese OA1 patients and found that foveal hypoplasia may be a key indicator of OA1 in patients with nystagmus of unknown cause. Identification of mutations in *GPR143* and more information regarding clinical manifestations should facilitate early diagnosis, appropriate early therapy, and genetic counseling for this disease.
